# Cervical stenosis in a professional rugby league football player: a case report

**DOI:** 10.1186/1746-1340-13-15

**Published:** 2005-08-03

**Authors:** Henry Pollard, Lotte Hansen, Wayne Hoskins

**Affiliations:** 1 Department of Health and Chiropractic, Macquarie Injury Management Group, Macquarie University, 2109, Sydney Australia; 2 Lotte Hansen Chiropractic, 70 Donald Street, Hamilton NSW 2303 Australia; 3 Department of Health and Chiropractic, Macquarie Injury Management Group, Macquarie University, 2109, Sydney Australia

**Keywords:** manipulation, chiropractic, non operative treatment, cervical, radiculopathy, sport, injury, rugby league

## Abstract

**Background:**

This paper describes a case of C7 radiculopathy in a professional rugby league player after repeated cervical spine trauma. The report outlines the management of the patient following an acute cervical hyperflexion injury with chiropractic manipulation and soft tissue therapies. It also presents a change in approach to include distractive techniques on presentation of a neurological deficit following re-injury. The clinical outcomes, while good, were very dependent upon the athlete restricting himself from further trauma during games, which is a challenge for a professional athlete.

**Case presentation:**

A 30-year old male front row Australian rugby league player presented complaining of neck pain after a hyperflexion and compressive injury during a game. Repeated trauma over a four month period resulted in radicular pain. Radiographs revealed decreased disc height at the C5-C6 and C6-C7 levels and mild calcification within the anterior longitudinal ligament at the C6-C7 level. MRI revealed a right postero-lateral disc protrusion at the C6-C7 level causing a C7 nerve root compression.

**Conclusion:**

Recommendations from the available literature at the present time suggest that conservative management of cervical discogenic pain and disc protrusion, including chiropractic manipulation and ancillary therapies, can be successful in the absence of progressive neurological deficit. The current case highlights the initial successful management of a football athlete, and the later unsuccessful management. This case highlights the issues involvement in the management of a collision sport athlete with a serious neck injury.

## Background

Effective cervical injury management requires an understanding of the pathomechanics of injury [1]. There are numerous reports in the literature on cervical injuries in full contact collision sports such as rugby (United Kingdom), rugby league/union (Australia) and American football (United States) [2-7]. However, there is a lack of literature on treatment, particularly non-operative manual therapy. This case report proposes an approach to address discogenic pain and disc protrusion. Injury management of a professional athlete is a challenge given the pressure on the player to play each match and perform through the competitive season.

## Case Presentation

### Case Report

A 30 year old professional Australian rugby league player presented complaining of diffuse neck pain following a hyperflexion and compressive neck injury while being tackled with his neck in maximal flexion. The injury occurred six days prior to presentation. There had been no significant previous history of injury or concussions over a 15 year career.

On examination, pain was reproduced in the right trapezius area on forward flexion and neurological testing was normal. There was no upper limb pain present. Other testing, including vertebral artery and blood pressure testing, proved unremarkable. Treatment consisted of high velocity, low amplitude manipulative therapy directed to the C2, C5 and T1 levels and proprioceptive neuromuscular facilitation (PNF) stretching to the trapezius and cervical musculature. This resulted in full pain free cervical ROM in three treatments.

Over the next four months the patient presented on five more occasions for similar cervical injuries following hyperflexion and compressive injuries while being tackled in matches. Symptoms progressed to include frontal headaches and cervico-thoracic pain. Treatment continued to include manipulation to the cervical and thoracic spine, PNF, soft tissue massage and trigger point therapy to the trapezius, sterno-cleido mastoid (SCM) and suboccipital musculature. This resulted in symptomatic relief within two to three treatments at each presentation. Over the four month period blood pressure and upper limb neurological status was monitored which proved to be unremarkable.

Four months after the initial presentation, the patient presented again following a combined hyperflexion with left lateral rotation injury after a collision in a game. Severe pain was immediately experienced over the right lower neck with pain radiating into the right upper trapezius region. Cervical radiographic images were ordered, including lateral, anterio-posterior, antero-posterior open mouth, oblique and functional views in flexion and extension. These views revealed decreased disc height at the C5-C6 and C6-C7 levels with mild osteophytic lipping. No definite instability was demonstrated. On the oblique view the intervertebral foramina appeared normal throughout the cervical spine.

A working diagnosis of an acute C6 disc lesion was established and the patient was advised to consult the team physician for advanced imaging to determine the state of any disc disruption. He continued to receive two treatments per week to manage his symptoms. Treatment was directed toward the management of the acute symptoms with ice, soft tissue therapy and manipulation to sites around (but not on) the lesion. The patient was also advised to commence a short course of non-steroidal anti-inflammatory medication (NSAIDs). Practitioners should be mindful of their recommendations as all medications must be deemed appropriate according to the governing sport's policy on sports doping.

After each game for a two month period symptoms progressively worsened until he presented with severe constant pain in the right upper trapezius region. This time the right triceps muscle strength was significantly reduced and the pain was relieved by elevating the right arm over his head and by cervical traction, indicating possible neural impingement. Other upper limb neurological testing, including upper limb deep tendon reflexes and sensory testing, was negative.

The clinical diagnosis was altered to a C6 radiculopathy. Treatment was altered to intermittent cervical traction and pulsed ultrasound therapy to the right trapezius area twice per week. Home advice included the use of an ice pack (20 minutes on the hour) and intermittent cervical traction three times a day. NSAID use was advised.

Two weeks later the patient presented with cervico-thoracic pain after being hit on the left side of his head while being tackled. Cervical ROM was full but painful in right rotation, flexion and extension. Upper limb neurological testing was unremarkable. A non-specific soft tissue sprain/strain diagnosis was given. Treatment involving cervical and thoracic manipulation and massage was again delivered with symptomatic relief. He was advised not to play rugby league until his symptoms had settled.

The patient continued to play and one month later began experiencing numbness in the second and third digits of his right hand. The Door Bell Sign over the right lower cervical area (pressure over the IVF at the antero-lateral aspect of the neck) reproduced the radicular symptoms. Other upper limb neurological testing was normal. The patient continued to receive intermittent cervical traction, gentle mobilisation of the cervical spine and manipulation to the upper thoracic area, along with massage therapy twice per week.

Four days later team medical staff referred the patient for magnetic resonance imaging (MRI). The MRI revealed a right postero-lateral disc protrusion at the C6-C7 level. The patient was strenuously advised not to play for three weeks, or to do upper body weight training. Two weeks later after following this advice the patient had regained full cervical ROM and pain was only reproduced on passive flexion and extension. He was still experiencing numbness in the second and third digits of the right hand. He began playing games again before finishing the season three weeks later.

Follow up two months post season showed no pain or restriction to cervical ROM. He was still experiencing some numbness in the second digit of the right hand, and the right triceps muscle was slightly weak. Whilst a new symptom, the mild nature of the tricep weakness was not considered serious, but it was to be monitored closely for any signs of deterioration. The player was informed of this approach and consented to it. Other neurological and orthopaedic tests were negative (shoulder and elbow range of motion, and Phalen's test). The athlete was advised to gradually build up his weight training and to maintain cervical flexibility with strengthening and stretching exercises.

The player was still reluctant to stop football and has since been advised by his orthopaedic surgeon to have discectomy surgery and to fuse the C6-C7 level, requiring six months of rehabilitation before returning to play. The player did not have the surgery and rested until the following year where he began the process all over again. He is now retired from football.

### Literature Review

This case outlines a series of cervical traumas producing neck, arm and head pain. The series of injuries involved forced flexion, compression and lateral deviation away from the painful side. This mechanism is in contrast to the mechanism of extension with lateral deviation towards the painful side as described in the majority of studies of neck injuries in American football and rugby [2,5,6], The clinical signs suggest a disc herniation following repeated trauma resulting in compression of the C7 nerve root.

There are several studies reporting chronic recurrent cervical nerve root neuropraxia (sometimes called "chronic burner syndrome"), in American football [5,6] and in rugby players [2]. This can commonly occur during blocking, tackling or engaging in a scrum. Chronic burner syndrome can be defined as:

1) a chronic recurrent neuropraxia or axonotmesis, or both, of a nerve root associated with prolonged weakness,

2) time loss from practice and games, and

3) recurrence [6].

Nerve root compression in the intervertebral foramina secondary to disc herniation or degenerative changes, or both, is the most common cause in football players seen with recurrent or chronic burners [6]. In such cases, degenerative changes frequently present with concurrent cervical canal stenosis and can predispose injury [8].

A correlation seems to exist between chronic recurrent cervical nerve root neurapraxia and cervical canal stenosis in tackled football players [1,5-7] and risk of more serious cervical spine injury increases with increasing stenosis [9]. A spinal canal-vertebral body ratio (Pavlov's ratio) on lateral radiographs of 0.80 or less (normal ratio 1:1) at one or more levels has been found in a tackle football population who have experienced an episode of cervical cord neuropraxia manifested by sensory and/or motor symptoms [10]. Despite a series of minor neurological insults, no correlation between the prodromal episodes of cord neuropraxia and occurrence of permanent quadriplegia has been found [10]. Also, the presence of uncomplicated developmental narrowing of the stable cervical spine does not predispose permanent neurological injury [1].

Absolute contraindications to continued participation in contact sports has been recommended to apply to those individuals who have had a documented episode of cervical cord neurapraxia associated with the following:

• ligamentous instability,

• intervertebral disc disease with cord compression,

• significant degenerative changes,

• MRI evidence of cord defects or swelling,

• positive neurological findings lasting more than 36 hours,

• more than one recurrence [10].

The extremely low predictive value of Pavlov's ratio (as an indicator of clinically relevant spinal stenosis) precludes its use as a screening mechanism for determining participation in contact activities [7]. To accurately assess spinal canal stenosis, cross-sectional imaging technology such as MRI, contrast positive CT, and myelography should be employed [7]. Plain radiographic identification of a narrow spinal canal in a player sustaining cervical cord neuropraxia warrants MRI investigation to rule out soft tissue based stenosis [11].

### Mechanism of Injury

Most of the literature on cervical spine injuries in football, such as burner syndrome, emphasises an extension type mechanism of injury. In our case, the mechanism of injury involved both hyperflexion and a compressive force. As hyperflexion involves more compressive load to the cervical spine than extension, this combination has a greater potential for injury, particularly if a stenosis situation concurrently exists [12].

With cervical hyperflexion, the spinolaminar line of the superior vertebra and the posterior superior aspect of the vertebral body below approximate, resulting in a rapid decrease of the spinal canal with compression of the spinal cord [1]. The brief, sudden deformation of the cord is thought to produce disturbed sensory and motor function below the involved level [1,13]. In most instances of acute spinal injury, disruption of cord function is the result of local cord anoxia and increased concentration of intracellular calcium [1]. Playing with improper technique, such as spear tackling, has been associated with catastrophic injuries [14]. In the case presented in this report, the technique of running at a tackler with neck hyperflexion before impact contributed to the repetitive history of injury and should have been corrected.

Hyperflexion injuries in Whiplash-Associated-Disorders (WAD) do not involve the exact same mechanism of injury (i.e. absence of axial compression) but the soft tissue damage can be very similar [15]. For example, Grade III WAD features include: cervical herniated disc, cervicalgia with headaches and limited range of motion combined with neurologic symptoms and signs are present [15].

With compression, a force exerted through the crown of the head can be transmitted through the skull to the cervical vertebrae resulting in the crushing of the vertebrae and extrusion of the vertebral body and disc material posteriorly into the cervical vertebral canal [3]. When the cervical spine is in hyperflexion with rotation, vertebral dislocation without fracture is possible, which is more likely if the head is locked on the ground adding a compressive force [15]. The most damaging mechanisms of injury to the spine are torsional and combined motions (i.e. forward flexion and lateral rotation) with a combined axial load [17,18].

### Management

Most of the literature involving treatment of patients with cervical disc herniations producing neurologic loss reports surgical outcomes. Furthermore, reports of patients with cervical spondylotic myelopathy show the symptoms progress gradually or step-wise and recovery of neurological function after conservative treatment or decompression surgery is common [19-21]. There is a lack of literature on manual therapy for an acute presentation of chronic recurrent cervical nerve root neuropraxia.

The treatment protocol in the acute phase should involve rest, ice and intermittent traction [22]. Case reports have shown successful chiropractic management of radicular and pseudoradicular pain through high-velocity, low amplitude thrust techniques and associated soft tissue therapies [23]. Manipulative therapy to the spinal segments above and below the painful levels, after the appropriate pre-manipulative provocation testing for verebrobasilar insufficiency has been performed, is believed to aid in increasing fluid infusion, and decreasing nociceptive input to the spinal cord [24]. The chiropractic literature suggests, in cases of cervical disc herniation, that manipulative therapy of the involved level is delivered only in the sub-acute phase, with the line of thrust being only in the pain-free directions [22].

The practitioner should be mindful of the potential for iatrogenic joint instability to occur. Damage to the supporting structures resulting in hypermobile joints can be aggravated by and result from repeated manipulations [25]. The recommended management protocol for Grade III WAD, which is a similar injury, is shown in Table 1 and could be viewed as a guideline for management of footballers with cervical stenosis [15].

**Table 1:** Management of Grade III Whiplash Associated Disorders (WAD) [15]

• Soft collars should not be used as they do not adequately immobilise the spine
• Narcotic analgesics may occasionally be needed for pain relief. Psychopharmacologic drugs and muscle relaxants should not be used
• In the few cases in which rest for the neck might be indicated, it should be limited to less than four days and followed by early activation
• The Task Force consensus is that manipulative treatments by trained persons for the relief of pain and facilitating early mobility can be used in WAD
• Long term repeated manipulation or physiotherapy without multidisciplinary evaluation is not justified
• Surgery for WAD patients is rarely indicated. Surgery is only indicated for Grade III patients with progressive neurologic deficit or persisting arm pain

The role of surgical versus non-surgical treatment of patients with cervical disc herniation has been compared in a longitudinal cohort study [26]. Twenty-six subjects with a clearly defined diagnosis of cervical herniated nucleus pulposus on MRI were evaluated for outcome with conservative treatment. This included ice, rest, hard cervical collar, NSAIDs for six to 12 weeks, manual and mechanical traction followed by home traction, and progressive strengthening exercises of the shoulder girdle and chest with training in postural control and body mechanics training. Follow up for over a year showed that 24 of the patients were successfully treated without surgery. Twenty patients achieved good or excellent results determined by symptom level, activity and function level, medication and ongoing medical care, job status and satisfaction. Only two patients required surgery suggesting that many cervical disc herniations can be successfully managed with conservative treatment.

Low level evidence from the available literature suggests that conservative management of discogenic pain and disc protrusion can be as successful as surgical treatment. However, such approaches, including corticosteroid injections [28], need to be validated by higher level evidence such as a randomised controlled trial [26,27]. Further, the current clinical trials have not included an athletic population.

It has been suggested that individuals with disc herniations will require lifelong management to ensure long-term participation in sport [26,27]. However, at present there is little evidence to support or refute such an approach. Based on a study of American football players the current recommendations for an athlete with intervertebral disc injury participating in a contact sport is as follows:

1. No contraindication to participate in contact sport in individuals with a healed disc herniation treated conservatively,

2. Relative contraindication in individuals with facet instability,

3. Absolute contraindication in individuals with:

a. Acute disc herniation (with or without neurological findings)

b. Acute or chronic disc herniation with decreased cervical range of motion,

c. Acute or chronic disc herniation with signs and symptoms of cord neuropraxia due to congenital stenosis of the cervical canal [4].

According to these recommendations the patient described in this case report would belong in group 3a) and should therefore not have played until his symptoms had subsided. With professional athletes a player can be reluctant to report injuries for fear of losing a spot in the team or losing wages. Despite not believing it is safe to play with injuries, many athletes are willing to risk doing so [29]. The authors of this study further concluded that: individuals with uncomplicated cervical cord neuropraxia can return to play without risking further damage, and various clinical manifestations are not related to the radiological findings.

The current literature agrees that the fundamental requirements for return to contact sports to include: normal strength, painless range of motion, a stable vertebral column and adequate space for the neurological elements [14]. Return to play guidelines need to be further investigated as several approaches exist and are open to interpretation. Some approaches allow a more liberal return to play criteria and would more likely be used with professional sports persons. Other return to play criteria are less liberal and would be used for amateurs and junior players

A one level surgical fusion has been suggested not to present any contraindication to participation in contact activities provided the athlete is completely asymptomatic and neurologically normal when commencing sport [4]. In a prospective study it was found that most cervical disc herniations regress with time and without the need for surgical resection [30]. Patients were finally examined and discharged from care because of sustained pain control at an average of six months. These findings are similar to four cases reporting spontaneous resolution of cervical herniation [31]. Given that surgery will require six months of rest from contact sport and a rehabilitation program, one may question whether the same outcomes with aggressive conservative therapy would have similar if not better results. Guidelines for surgical cases need to be clearly defined and randomised controlled trials comparing surgical to conservative care performed.

A review of the management of lumbar intervertebral disc injuries in athletes suggests that the high recurrence rate of low back pain may indicate that the resolution of symptoms is accompanied by restoration of function [27]. This is possibly due to functional changes that occur with injury, which can be assumed to occur with cervical disc injuries. Cervical dysfunction can be caused by failure to rehabilitate previous injuries [32,33], a scenario commonly encountered in the professional athlete who frequently returns to play sooner than the ideal. This becomes important for an athlete as performance can suffer in the absence of pain but in the presence of subtle biomechanical maladaptations [27].

## Conclusion

This case report has outlined the progression of cervical injury to a disc protrusion resulting in a C7 radiculopathy in a professional rugby league player, due to numerous blows to the cervical spine after a series of hyperflexion injuries. The patient ultimately suffered a severe forced flexion combined with left lateral flexion injury to the cervical spine and experienced sensory and motor changes in the right C7 nerve root distribution. When it became apparent that there was intervertebral foramen encroachment secondary to a disc protrusion the treatment protocol changed toward a more conservative approach.

It is important that the athlete is informed of the problem with particular regard to potential risks if they continue to play. When considering surgery, the long-term consequences of the intervention should be thoroughly discussed with the athlete along with all potential management options including no treatment and retirement from the sport. Further research is needed on the chiropractic management of acute athletic injuries to the spine and the long-term outcome for surgery versus conservative management of patients wishing to continue their athletic career.

## Authors' contributions

HP conceived of the study, participated in its design and helped to draft and edit the manuscript.

LH provided treatment to the subject and helped draft the manuscript

WH helped to collect literature and draft the manuscript.

All authors read and approved the manuscript.
